# Efficacy and safety of two interleukin-17 inhibitors (bimekizumab and secukinumab) in the management of hidradenitis suppurativa: a systematic review and meta-analysis of randomized controlled trials

**DOI:** 10.1093/skinhd/vzag102

**Published:** 2026-07-10

**Authors:** Hana Ishkirat, Yozahandy Angeles Abarca-Pineda, Arwa Ahmed, Fatima Shamim, Tala Elremmash, Bilal Haider Malik, Moaz Mohsin, F N U Deeksha, Teenu Maria Jeswin, Amandeep Kaur, Asma’a Munasar Ali Alsubari, Muhammad Ayyan

**Affiliations:** Department of Medicine, Arab American University, Ramallah, Palestine; Department of Internal Medicine, Medica Sur, Mexico City, Mexico; Department of Medicine, University of Sharjah, Sharjah, UAE; Department of Medicine, Tbilisi State Medical University, Tbilisi, Georgia; Department of Medicine, Istinye University, Istanbul, Turkey; Department of Dermatology, Betsi Cadwaladr University Health Board, Bodelwyddan,UK; Department of Acute Medicine, Royal Stroke University Hospital, Stoke-on-Trent, UK; Department of Medicine, Government Medical College, Patiala, India; Department of Medicine, Doncaster Royal Infirmary, Doncaster, UK; Department of Medicine, Government Medical College, Patiala, India; Faculty of Medicine, Sana’a University, Sana’a, Yemen; Department of Medicine, King Edward Medical University, Lahore, Pakistan

## Abstract

**Background:**

Hidradenitis suppurativa (HS) is a chronic inflammatory skin condition that primarily affects the intertriginous regions, causing manifestations such as inflammatory nodules, abscesses and scarring.

**Objectives:**

To systematically evaluate the efficacy and safety of interleukin (IL)-17 inhibitors, including bimekizumab and secukinumab, for the treatment of moderate-to-severe HS based on evidence from randomized controlled trials (RCTs).

**Methods:**

We searched various electronic databases, including MEDLINE (via PubMed), Embase and CENTRAL to retrieve RCTs on the use of two IL-17 inhibitors (bimekizumab and secukinumab) for the management of HS. Dichotomous outcomes were reported as relative risk (RR) with 95% confidence intervals (CIs), and continuous outcomes were reported as mean difference (MD) with 95% CIs.

**Results:**

Our meta-analysis included 5 RCTs with a total of 2188 patients. According to our analysis, IL-17 inhibitors significantly increased the proportion of patients with HiSCR50 (Hidradenitis Suppurativa Clinical Response) (RR 1.45, 95% CI 1.26–1.66) and HiSCR75 (RR 2.03, 95% CI 1.42–2.90). IL-17 inhibitors significantly increased the proportion of patients with Hidradenitis Suppurativa Symptom Daily Diary (HSSDD) worst skin pain response (RR 2.46, 95% CI 1.41–4.30). The IL-17 inhibitor group was associated with a statistically significant decrease in HSSDD worst skin pain score from baseline compared with the placebo group (MD −1.02, 95% CI −1.44 to −0.60). The intervention group was also associated with a statistically significant decrease in Dermatology Life Quality Index score from baseline compared with the placebo group (MD −1.77, 95% CI −2.54 to −0.99). The treatment-emergent adverse events were similar in both groups.

**Conclusions:**

IL-17 inhibitors are associated with an improvement in clinical response and pain outcomes in patients with moderate-to-severe HS, with an acceptable safety profile in the available studies. Given the limited number of trials with no head-to-head comparisons, further large-scale and independent studies are required to clarify their long-term role, particularly in patients whose HS is refractory to conventional and biologic therapies.

## Teaser

IL-17 inhibitors (bimekizumab and secukinumab) demonstrate significant efficacy in improving clinical response (HiSCR50/75), reducing pain and enhancing quality of life in moderate-to-severe hidradenitis suppurativa, with a favourable safety profile. The encouraging results pertaining to IL-17 inhibitors can set a new standard of care, in particular for those patients who have failed conventional therapies, including TNF-α inhibitors.

What is already known about this topic?

- Hidradenitis suppurativa (HS) is a chronic inflammatory skin disease associated with substantial pain, impaired quality of life and limited therapeutic options.
- Interleukin (IL)-17 signalling is implicated in the pathogenesis of HS, and IL-17 inhibitors have emerged as potential therapeutic agents.
- Individual randomized trials have reported promising outcomes with bimekizumab and secukinumab, but the overall evidence has not been comprehensively synthesized.

What does this study add?

- This meta-analysis of 5 RCTs involving 2188 patients demonstrates that IL-17 inhibitors significantly improve HiSCR50, HiSCR75 and pain-related outcomes compared with placebo.
- Treatment with IL-17 inhibitors is associated with improvements in quality of life and reductions in skin pain scores.
- The available evidence suggests a favourable safety profile, supporting IL-17 inhibition as an effective treatment strategy for moderate-to-severe HS.

Hidradenitis suppurativa (HS), also known as acne inversa, is a chronic inflammatory skin disorder that affects the folliculo­pilosebaceous units (FPSUs), particularly the follicular portion of FPSUs, causing follicular occlusion.^1^ The areas most commonly affected are intertriginous regions, such as the axilla, groin and perineal areas. Early manifestations include inflammatory nodules and abscesses, while advanced stages are characterized by skin tunnels, comedones and scarring.^2,3^ The prevalence of HS has been studied in a few epidemiological studies, and it ranges from less than 1% to 4%.^4,5^ In the USA, the incidence is estimated at 11.4 cases per 100 000 individuals, while in the UK, the prevalence is approximately 0.77%. According to recent data from the Global Hidradenitis Suppurativa Atlas, prevalence estimates for HS remain highly variable, ranging from as low as 0.05% (based on insurance-claims databases) to as high as 4.1% in certain in-­person dermatological screenings.^6^ Notably, HS is more than twice as prevalent in women and three times more common in African American people, as indicated by a US retrospective study.^7^

The management strategies of HS include medical and surgical approaches.^8,9^ Mild cases of HS are often treated with topical agents such as clindamycin, while patients with moderate-to-­severe HS typically require systemic therapies, including antibiotics, corticosteroids and biologic agents. Biologics approved for HS include adalimumab and infliximab [tumour necrosis factor (TNF)-α], as well as secukinumab and bimekizumab [interleukin (IL)-17 inhibitors]. Surgical interventions are generally reserved for advanced disease with irreversible tissue damage. However, surgical therapy, particularly procedures such as deroofing, is increasingly recognized as an important adjunct to medical therapy and may be employed across a range of disease severities, including patients with localized tunnels or nodules.

The role of IL-17 and its receptors in the pathogenesis of HS has been well studied. Studies have shown elevated expression of IL-17 and its receptors in HS skin lesions. IL-17 promotes the release of inflammatory mediators such as antimicrobial peptides, chemokines, growth factors and TNF, which recruit inflammatory cells like neutrophils, macrophages and lymphocytes, exacerbating the disease.^10–12^

Adalimumab was the first biologic agent approved for the management of HS by the US Food and Drug Administration (FDA) and the European Medicines Agency (EMA). Despite the availability of biologic therapies such as TNF-α inhibitors (e.g. adalimumab and infliximab), a significant number of patients with HS either do not respond adequately to these treatments or experience partial relief, underscoring the need for alternative therapeutic options. IL-17 inhibitors, such as secukinumab and bimekizumab, have emerged as promising candidates due to their ability to target this specific inflammatory pathway. Secukinumab, a monoclonal ­antibody targeting IL-17A, has also received approval from the EMA, FDA and the UK Medicines and Healthcare products Regulatory Agency for the treatment of moderate-to-severe HS.^13^ Bimekizumab, a humanized monoclonal antibody that inhibits IL-17A, IL-17F and IL-17AF, thus preventing their binding to IL-17RA/IL-17RC receptors, is currently approved for the treatment of psoriasis and psoriatic arthritis.^14^ Recent data from two randomized controlled trials (RCTs) have demonstrated its efficacy in HS, leading to EMA approval, although FDA approval is still pending.^15^ Although biologic therapies such as bimekizumab have shown promise in clinical trials, national guidance for use within the UK remains limited: according to the National Institute for Health and Care Excellence, bimekizumab is not currently recommended for HS due to uncertainty about its cost-effectiveness and lack of a sustainable reimbursement route.^16^

Our study aimed to comprehensively analyse the efficacy and safety of two IL-17 inhibitors (secukinumab and bimekizumab) for the management of HS by pooling data from all published RCTs.

## Materials and methods

### Protocol and registration

This review was conducted and is reported in accordance with the guidelines of the Cochrane Handbook for Systematic Reviews and PRISMA statement.^17,18^ The review has been registered with PROSPERO (CRD42024593019).

### Eligibility criteria

Our eligibility criteria were defined using the Population–­Intervention–Comparator–Outcome–Study (PICOS) model. Accordingly, studies were included in our review if they met the following inclusion criteria: (i) Population: patients with HS; (ii) Intervention: IL-17 inhibitors (bimekizumab or secukinumab); (iii) Comparator: placebo or any active comparator; (iv) Outcomes: studies reporting at least one primary outcome; and (v) Study: RCTs only. Other study designs, like case series, case reports, cohort studies and nonrandomized trials, were excluded.

### Literature search and search strategy

We systematically searched the following databases and trial registers from inception to January 2025 to identify studies evaluating the safety and efficacy of the IL-17 inhibitors bimekizumab and secukinumab in patients with HS: Cochrane Central Register of Controlled Trials (CENTRAL, via the Cochrane Library), MEDLINE (via PubMed), Embase (via Ovid), ClinicalTrials.gov, ProQuest Dissertations and Theses Global (PQDT), and OpenGrey. The reference lists of relevant studies were searched, and forward citation searching was also conducted to retrieve relevant studies.

### Study selection process

All articles retrieved from our online search were screened and deduplicated using Rayyan (https://www.rayyan.ai/). Using the eligibility criteria mentioned above, the retrieved articles were primarily screened based on their titles and abstracts by two review authors independently. This was followed by a secondary screening, which involved a comprehensive review of the full-text articles to assess their eligibility. This was also conducted by two review authors independently. Any disagreements between the two reviewers were resolved by discussion between the two authors or by a third author.

### Data extraction

Following screening, the data from all included RCTs was extracted into a prepiloted Microsoft Excel spreadsheet. The study characteristics, population characteristics, intervention and comparator details, and outcomes were extracted by two review authors independently. Any disagreements between the two reviewers were resolved by discussion between the two authors or by a third author.

### Outcomes

Our primary outcomes were Hidradenitis Suppurativa Clinical Response (HiSCR50), defined as a 50% decrease in abscess and nodule count at 16 weeks. The secondary outcomes included HiSCR75 (75% decrease in abscess an nodule count), the Hidradenitis Suppurativa Symptom Daily Diary (HSSDD) worst skin pain response, HSSDD worst pain score change from baseline, change in Dermatology Life Quality Index (DLQI) score from baseline and adverse events.

### Risk of bias assessment

We assessed the risk of bias in the included studies using the revised Cochrane Risk of Bias tool for randomized trials (RoB 2.0), which assesses bias in the following five domains: (i) bias arising from the randomization process; (ii) bias caused by deviations from intended interventions; (iii) bias caused by missing outcome data; (iv) bias in the measurement of the outcome; and (v) bias in the selection of the reported result. All the authors independently rated the risk of bias for each included study as low, high or some concern. Any disagreement was resolved by a third reviewer.

### Data synthesis

We used Review Manager (RevMan, version 5.4) for data analyses. Dichotomous outcomes were reported as relative risk (RR) with 95% confidence intervals (CIs). Continuous outcomes were reported as mean difference (MD) with 95% CIs. The Der-Simonian and Laird random-effects model was used to perform meta-analyses. For each analysis, we calculated the I^2^ statistic to assess the clinical heterogeneity among studies. We conducted a subgroup analysis of primary outcome based on the frequency of administration of the IL-17 inhibitor (every 2 weeks vs. every 4 weeks) and the type of IL-17 inhibitor (bimekizumab vs. secukinumab).

For trials with multiple IL-17 inhibitor dosing arms sharing a common placebo group, we followed Cochrane Handbook recommendations to avoid unit-of-analysis errors. When clinically appropriate, multiple intervention arms were combined into a single group by summing event counts and total participants for dichotomous outcomes, and by calculating pooled means and standard deviations for continuous outcomes. These procedures ensured that each participant contributed only once to any individual meta-analysis comparison.

### Certainty of evidence assessment

Two authors (H.I. and M.A.) independently evaluated the certainty of the evidence using the five GRADE domains: study limitations, consistency of effect, imprecision, indirectness and publication bias.^19^ Pooled estimates were considered imprecise if the optimal information size was not met or if the 95% CIs included both the null effect and clinically important benefit or harm.^20^ The overall quality of evidence was categorized as high (further research is very unlikely to change our confidence in the effect estimate), moderate (further research may have an important impact on confidence and could change the estimate), low (further research is likely to have an important impact and may change the estimate) or very low (effect estimate is very uncertain). Results are presented in a summary of findings in Table 1.

**Table 1:** GRADE summary of findings

Outcome	No. of participants (studies)	Effect estimate (95% CI)	Risk of bias	Inconsistency	Indirectness	Imprecision	Publication bias	Quality of evidence (GRADE)
HiSCR50	2156 (5)	1.45 (1.26–1.66)	Not serious	Not serious	Not serious	Serious	Undetected	⊕⊕⊕⊖
								Moderate
HiSCR75	1072 (3)	2.03 (1.42–2.90)	Not serious	Not serious	Not serious	Serious	Undetected	⊕⊕⊖⊖
								Low
HSSDD worst skin pain response	1014 (2)	2.46 (1.41–4.30)	Not serious	Not serious	Not serious	Serious	Suspected	⊕⊕⊖⊖Low

CI, confidence interval; HiSCR50/75, Hidradenitis Suppurativa Clinical Response 50/70; HSSDD, Hidradenitis Suppurativa Symptom Daily Diary.

## Results

A total of 353 studies were retrieved upon searching the electronic databases. After the screening process, three published studies reporting data from five RCTs were included in our meta-analysis.^13,15,21^ The PRISMA flowchart of the study selection process is presented as Figure 1.

**Figure 1:**
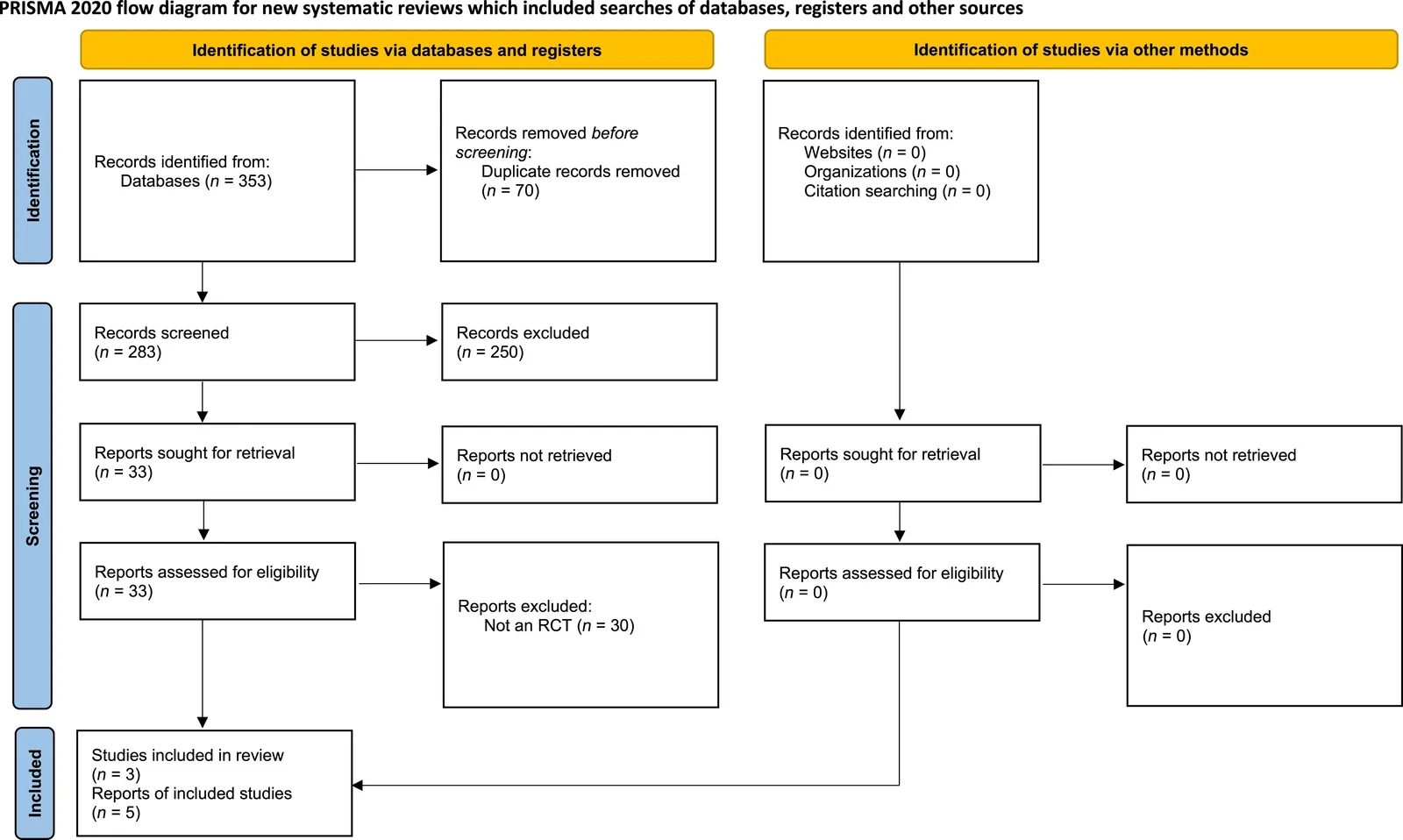
PRISMA flow chart of included and excluded trials. RCT, randomized controlled trial.

There were a total of 2188 patients included in our study. Bimekizumab was the intervention drug in three RCTs, while in the other two RCTs, it was secukinumab. While all included studies evaluated bimekizumab and secukinumab, each trial included multiple dosing arms to assess different schedules. Therefore, doses were not fully consistent across all participants, and outcomes are presented according to the specific dosing regimens evaluated in each study. The duration of intervention ranged from 12 to 16 weeks. The mean age of all participants was 35.5 years. The study and patient characteristics of the included studies are presented in Table 2.

**Table 2:** Characteristics of the included studies

Study ID	Location	Time period (weeks)	Follow-up period (weeks)	Number of patients	Intervention	Control	Duration of intervention (weeks)	Mean age (years) (every 2 weeks vs. every 4 weeks vs. placebo)	Male sex (%) (every 2 weeks vs. every 4 weeks vs. placebo)	Previous use of biologic therapy (every 2 weeks vs. every 4 weeks vs. placebo)	Disease duration, years (every 2 weeks vs. every 4 weeks vs. placebo)	Hurley stage 2 (every 2 weeks vs. every 4 weeks vs. placebo)	Hurley stage 3 (every 2 weeks vs. every 4 weeks vs. placebo)
BE HEARD 1 2024^15^	Multicentre (86 locations)	48	20	289 vs. 144 vs. 72	Bimekizumab 320 mg	Placebo: 1 mL prefilled syringe of 0.9% sodium chloride aqueous solution	16	36.0 vs. 35.0 vs. 33.5	39 vs. 32 vs. 39	76 vs. 31 vs. 19	5.7 vs. 5.6 vs. 8.7	149 vs. 71 vs. 34	140 vs. 73 vs. 38
BE HEARD 2 2024^15^	86 sites	48	20	291 vs. 144 vs. 74	Bimekizumab 320 mg	Placebo	16	35 vs. 33 vs. 37	48 vs. 46 vs. 58	41 vs. 16 vs. 10	4.9 vs. 4.2 vs. 4.8	177 vs. 89 vs. 45	114 vs. 55 vs. 29
Glatt 2021 (intervention vs. control)^21^	North America, Europe, Asia Pacific	34	20	46 vs. 22	Bimekizumab 320 mg	Placebo and adalimumab 40 mg every week	12	37.4 vs. 40.7	35 vs. 33	NR	9 vs. 9.5	23 vs. 10	23 vs. 11
SUNSHINE 2023^13^	40 countries	52	16	181 vs. 180 vs. 180	Secukinumab 300 mg	Placebo	16	37.1 vs. 35.7 vs. 35.5	44 vs. 44 vs. 43	44 vs. 39 vs. 46	7.4 vs. 6.6 vs. 7.5	104 vs. 107 vs. 121	70 vs. 63 vs. 51
SUNRISE 2023^13^	40 countries	52	16	180 vs. 180 vs. 183	Secukinumab 300 mg	Placebo	16	37.3 vs. 35.5 vs. 36.2	46 vs. 43 vs. 43	36 vs. 42 vs. 48	7.1 vs. 8.2 vs. 7.0	92 vs. 106 vs. 110	82 vs. 68 vs. 70
NR, not reported.													

### Risk of bias

All five RCTs had a low risk of bias, according to the Cochrane risk of bias tool (RoB 2.0). The risk of bias figure is presented as Figure S1 (see Supporting Information).

### Outcomes

#### HiSCR50

Our pooled analysis of data from five RCTs showed that IL-17 inhibitors significantly increased the proportion of patients achieving HiSCR50 (RR 1.45, 95% CI 1.26–1.66). The heterogeneity among studies was minimal (I^2^ = 0%) (Figure 2). This represents a clinically meaningful absolute increase in response compared with placebo. The observed low heterogeneity may reflect the inclusion of RCTs with broadly similar study designs, patient populations with moderate-to-severe HS (Hurley stage II–III), comparable outcome measures and relatively consistent dosing regimens across studies. The overall quality of evidence was rated as moderate due to concerns about inconsistency (Table 1).

**Figure 2:**
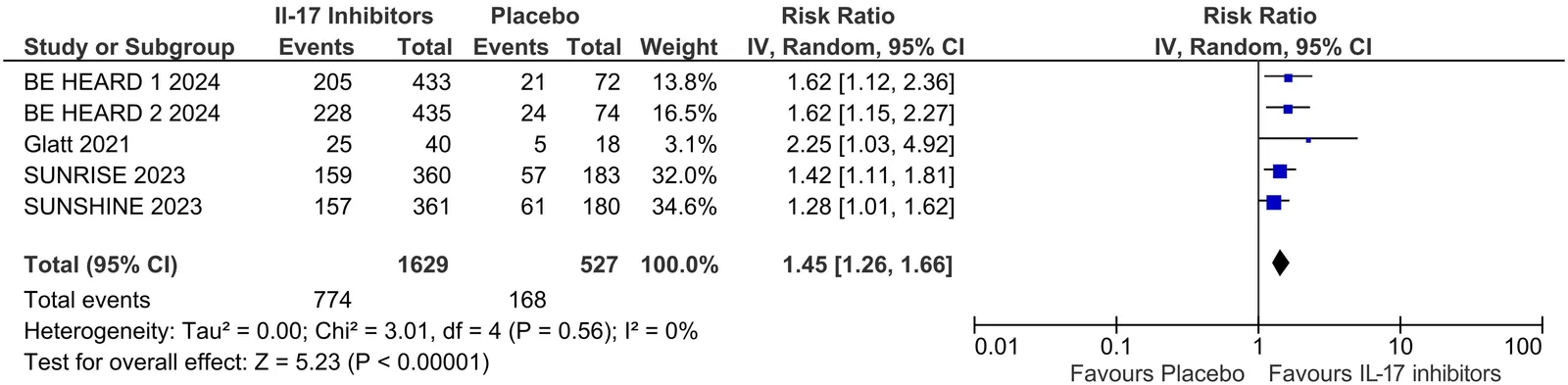
Comparison of HiSCR50 between patients receiving IL-17 inhibitors or placebo. CI, confidence interval; HiSCR50, Hidradenitis Suppurativa Clinical Response 50; IL, interleukin; IV, inverse variance.

#### HiSCR75

The IL-17 inhibitor group was associated with a statistically significant increase in patients achieving HiSCR75 compared with the placebo group (RR 2.03, 95% CI 1.42–2.90). The heterogeneity among studies was minimal (I^2^ = 0%) (Figure S2; see Supporting Information). The overall quality of evidence was rated as low due to concerns about inconsistency and publication bias (Table 1).

#### HSSDD worst skin pain response

Our pooled data analysis from two RCTs showed that IL-17 inhibitors significantly increased the proportion of patients with HSSDD worst skin pain response (RR 2.46, 95% CI 1.41–4.30). The heterogeneity among studies was minimal (I^2^ = 0%) (Figure S3; see Supporting Information). The overall quality of evidence was rated as low due to concerns about inconsistency and publication bias (Table 1).

#### DLQI score change from baseline

The IL-17 inhibitor group was associated with a statistically significant decrease in DLQI score from baseline compared with the placebo group (MD −1.77, 95% CI −2.54 to −0.99). The heterogeneity among studies was low (I^2^ = 9%) (Figure S4; see Supporting Information).

#### HSSDD worst skin pain score change from baseline

The IL-17 inhibitor group was associated with a statistically significant decrease in HSSDD worst skin pain score from baseline compared with the placebo group (MD −1.02, 95% CI −1.44 to −0.60). The heterogeneity among studies was moderate (I^2^ = 47%) (Figure S5; see Supporting Information).

### Adverse events

The incidence of overall treatment-emergent adverse events (TEAEs) was not significantly different between IL-17 inhibitors and placebo. However, the IL-17 inhibitor group was associated with a statistically significant increase in the incidence of drug-related TEAEs compared with the placebo group (RR 2.21, 95% CI 1.49–3.27). The heterogeneity among studies was minimal (I^2^ = 0%). These events were predominantly mild-to-moderate in severity. The IL-17 inhibitor group was associated with a significant decrease in the incidence of flares (RR 0.73, 95% CI 0.58–0.91; I^2^ = 22%). Both groups had similar incidences of headaches, fungal infections, hidradenitis, any TEAE, serious TEAE, *Candida* infections, discontinuations due to TEAE, infections and infestations, and hypersensitivity reactions. Fungal and *Candida* infections occurred more frequently in the IL-17 inhibitor group numerically; however, these differences did not reach statistical significance [fungal infections: RR 3.37 (95% CI 0.95–11.91); *Candida* infections: RR 2.53 (95% CI 0.49–13.19)], with wide CIs reflecting limited event numbers. The RRs of all the analyses of adverse events are presented in Table 3.

**Table 3:** Summary of effect measures of adverse events

*Outcome*	*Studies*	*Risk ratio (95% CI)*
*Flare*	3	0.73 (0.58, 0.91)
*Headache*	5	1.11 (0.75, 1.64)
*Fungal infections*	4	3.37 (0.95, 11.91)
*Any TEAE*	5	1.01 (0.94, 1.09)
*Discontinuation due to TEAE*	5	1.44 (0.60, 3.48)
*Drug*-*related TEAE*	3	2.21 (1.49, 3.27)
*Candida infections*	4	2.53 (0.49, 13.19)
*Serious TEAE*	5	0.96 (0.56, 1.66)
*Hidradenitis*	4	0.61 (0.36, 1.04)
*Hypersensitivity reactions*	4	1.38 (0.77, 2.47)
*Infections and infestations*	5	1.24 (0.93, 1.65)

CI, confidence interval; TEAE, treatment-emergent adverse event.

### Subgroup analyses

A subgroup analysis of the outcome HiSCR50 based on treatment frequency (every 2 weeks vs. every 4 weeks) and type of IL-17 inhibitor (bimekizumab vs. secukinumab) revealed no subgroup differences, with *P*-values of 0.85 and 0.15, respectively (Figures S5, S6; see Supporting Information). Similarly, a subgroup analysis of the outcome HiSCR50 based on the type of IL-17 inhibitor (bimekizumab vs. secukinumab) showed no subgroup differences, with a *P*-value of 0.15 (Figure 3). No significant differences were observed in subgroup analyses; however, the trials were probably underpowered to detect such differences given the small sample sizes. Therefore, these findings should be interpreted with caution.

**Figure 3:**
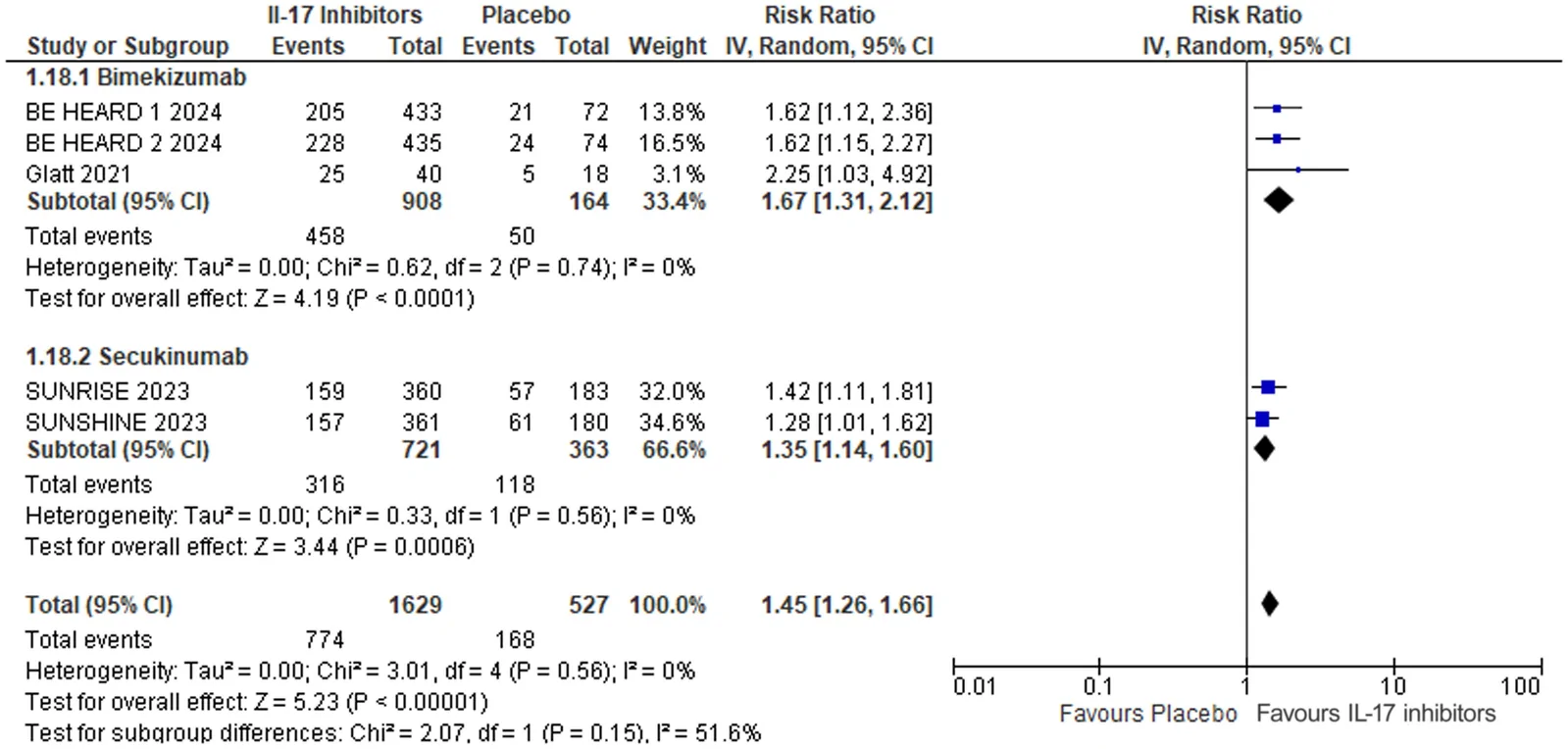
Comparison of Hidradenitis Suppurativa Clinical Response 50 (HiSCR50) between patients receiving interleukin (IL)-17 inhibitors or placebo according to type of IL-17 inhibitor. CI, confidence interval; IV, inverse variance.

## Discussion

In this meta-analysis of five RCTs, we demonstrate the clinical efficacy of two IL-17 inhibitors (bimekizumab and secukinumab) for the management of HS. The results revealed that IL-17 inhibitors significantly improved the likelihood of achieving key clinical responses. The 2-week and 4-week dosing regimens both led to higher rates of HiSCR50 and HiSCR75 compared with placebo, indicating meaningful improvements in disease control. Notable pain relief was observed, which resulted in a significant reduction in the HSSDD worst pain scores from baseline. Additionally, IL-17 inhibitors demonstrated favourable safety profiles, with no significant increase in TEAEs relative to placebo. In the included trials, fungal and *Candida* infections were generally mild-to-moderate in severity, manageable with standard antifungal therapy and rarely resulted in treatment discontinuation. Overall, IL-17 inhibitors demonstrated significant clinical efficacy in inducing treatment responses and reducing pain, with a favourable safety profile that supports their use as a viable therapeutic option for patients with moderate-to-severe HS (Hurley stage II–III).

Our findings are in agreement with the literature concerning the effectiveness and safety of IL-17 inhibitors in the treatment of inflammatory skin disorders like psoriasis and HS.^15,22–24^ The findings are consistent with previous studies, including the SUNSHINE and SUNRISE trials and BE HEARD 1 and 2 trials, which established the efficacy of secukinumab and bimekizumab, respectively, in HS through significant improvements in HiSCR50 and HiSCR75 rates compared with placebo.

Research has shown that both IL-17A and IL-17F contribute to HS pathology by promoting the production of inflammatory cytokines and chemokines in keratinocytes and immune cells.^25^ Additionally, recent reviews have emphasized bimekizumab’s efficacy demonstrated in phase III trials, which has supported its approval for HS treatment in some regions.^26^ A network meta-­analysis further confirmed that bimekizumab has effectiveness comparable to adalimumab in attaining HiSCR and improvement in quality of life (QoL) without an associated increase in adverse events compared with placebo.^27^ Although both bimekizumab and secukinumab target the IL-17 pathway, their mechanisms of action differ. Secukinumab selectively inhibits IL-17A, while bimekizumab blocks both IL-17A and IL-17F, cytokines that may independently contribute to inflammatory signalling.

A systematic review has highlighted that baseline disease severity can impact the efficacy of IL-17 inhibitors in HS treatment.^24^ Furthermore, high levels of IL-17, especially IL-17A and IL-17F, relate to the more severe stages of HS, such as Hurley stage III.^28^ This baseline inflammatory activity predicts the therapeutic response to IL-17 inhibition, with therapies such as secukinumab demonstrating enhancements in clinical scores and pain reduction in these severe cases.

Additionally, improvements in QoL from the DLQI scores also concur with the available literature evidence of Glatt *et al*.,^21^ who reported significant QoL benefits for patients bearing inflammatory conditions treated with IL-17 inhibitors. In particular, IL-17 inhibitors such as secukinumab, brodalumab and ixekizumab have shown remarkable reductions in symptoms associated with HS and simultaneously improved QoL measures, including DLQI score. Patients have reported better pain management and fewer skin lesions, which directly translated into increased daily functioning and social interactions.^24^ Studies have also shown that IL-17 inhibitors may result in a significant improvement in QoL as early as 4 weeks of treatment. Brodalumab, for example, has been associated with rapid reductions in pain, itch and depression, significantly improving patient-reported outcomes within short periods.^29^ Sustained use of IL-17 inhibitors, such as secukinumab, has likewise shown the clinical and QoL benefits to last for extended periods, with some studies continuing to report steady improvements in QoL scores for up to 136 weeks.^30^ Such findings suggest durable effects that could alleviate the chronic burden of HS.

In terms of safety, this meta-analysis found that IL-17 inhibitors were well tolerated, with adverse event rates comparable to placebo, findings that are consistent with safety data from the SUNSHINE, SUNRISE and Glatt *et al*. studies, which reported minimal safety concerns with secukinumab and bimekizumab. This meta-analysis reinforces the position of IL-17 inhibitors as effective and safe treatment options for moderate-to-severe HS, particularly for those who fail to respond to TNF-α inhibitors, including adalimumab. The enhanced clinical responses noted with IL-17 inhibitors, especially in attaining deeper results such as HiSCR75, highlight their potential to emerge as a preferred therapeutic choice in the treatment of HS. The observed numerical increase in fungal and *Candida* infections is biologically plausible, given the central role of the IL-17 pathway in muco­cutaneous host defence against fungal pathogens, particularly *Candida* species. IL-17 signalling promotes neutrophil recruitment and antimicrobial peptide production, which are critical for antifungal immunity. Importantly, in the included trials, these infections were predominantly mild-to-moderate in nature, manageable with standard therapy, and did not lead to a significant increase in serious adverse events or treatment discontinuation. Nevertheless, clinicians should remain vigilant for mucocutaneous fungal infections when prescribing IL-17 inhibitors.

The strengths of our meta-analysis include the strict adherence to Cochrane’s methodology, low statistical heterogeneity among the included studies and the inclusion of data from only RCTs, thus minimizing bias associated with observational studies. We tried to conduct subgroup analyses based on the treatment frequency and type of IL-17 inhibitor but did not find any significant difference between the groups. Another strength of our study was the low risk of bias in all the included studies.

Some limitations of our analysis must be acknowledged. Firstly, only five RCTs were included in the meta-analysis. This leads to low statistical power. Secondly, while statistical heterogeneity was low, clinical and methodological heterogeneity was present among trials due to differences in study design, population and underlying disease severity. The variation in underlying disease severity could play a role in the response to a specific drug. Thirdly, the follow-up duration of the trials was short, which limits the ability to determine long-term efficacy and safety profile. Furthermore, HS itself usually does not exist on its own, it tends to be accompanied by other comorbidities, which cannot be controlled in the trials, thus causing potentially less favourable results due to possible interaction of the biologics with the system. The ‘rescue systemic antibiotic’ therapy, which was used in all five trials, posed a significant limitation for the participants who had to take antibiotic therapy due to flare-ups. Those participants were then termed as ‘nonresponders’, and their results, despite having positive outcomes, were not included in the final data. All included studies were ­industry-sponsored, which represents a potential source of bias. A limitation of the included trials is the selection of patients with fewer comorbidities and overall better health status than typically seen in clinical practice. Consequently, the observed efficacy of IL-17 inhibitors may be higher in these trials than what might be achieved in real-world populations with multiple comorbidities. Finally, the lack of an active comparator and direct comparison with other biologics precludes definitive conclusions regarding their relative effectiveness. Although pooling across dosing regimens increased statistical power, it may obscure potential dose–response relationships. The included trials evaluated therapeutic dosing strategies; however, differential efficacy or safety between doses cannot be fully excluded and warrants further investigation.

Our study has numerous implications in clinical practice and research. Our analysis supports the use of both bimekizumab and secukinumab in treating moderate-to-severe HS. These agents should be considered in patients who do not respond to or are intolerant to the first-line medications, such as adalimumab. Due to the low statistical power and clinical heterogeneity among studies, our results should be interpreted with caution. IL-17 inhibitors, while effective, are high-cost therapies, which may limit access, particularly in low- and middle-income countries. Cost and insurance coverage should be considered when implementing these treatments in clinical practice.

Larger, multicentre RCTs, with longer follow-ups, are needed to better ascertain the long-term efficacy and safety of secukinumab and bimekizumab for the management of HS. The trials should focus on comparison with active comparators like other biologics. Future studies should investigate combination therapies using IL-17 inhibitors combined with other therapeutic interventions such as antimicrobials, hormonal treatments, biologics or surgical interventions for more complete disease control.

IL-17 inhibitors demonstrate improvement in clinical response and pain outcomes in patients with moderate-to-severe HS, with an acceptable safety profile in available studies. Given the limited number of trials with no head-to-head comparisons, further large-scale and independent studies are required to clarify their long-term role, particularly in patients refractory to conventional and biologic therapies.

## Supplementary Material

vzag102_Supplementary_Data
