# Supplementary material for: Efficacy and safety of two interleukin-17 inhibitors (bimekizumab and secukinumab) in the management of hidradenitis suppurativa: a systematic review and meta-analysis of randomized controlled trials
Source: Skin Health Dis. 2026 Jul 10;6(5):712–21. doi: 10.1093/skinhd/vzag102 (PMC13623431; doi:10.1093/skinhd/vzag102)
Supplement: Supplementary file 1 [file vzag102_supplementary_data.docx]

# SUPPLEMENTARY FILE

**Efficacy and Safety of Two IL-17 Inhibitors (Bimekizumab and Secukinumab) in the Management of Hidradenitis Suppurativa: A Systematic Review and Meta-Analysis of Randomized Controlled Trials (RCTs)**

**Figure S1. Risk of bias analysis of included studies using Cochrane’s Risk of bias tool (RoB 2.0)**

**Figure S2. Comparison of HiSCR75 between patients receiving IL-17 inhibitors or placebo. IV, inverse variance**

**Figure S3. Comparison of HSSDD worst skin pain response between patients receiving IL-17 inhibitors or placebo. IV, inverse variance**

**Figure S4. Comparison of DLQI score change from baseline between patients receiving IL-17 inhibitors or placebo. IV, inverse variance**

**Figure S5. Comparison of HSSDD worst skin pain change from baseline between patients receiving IL-17 inhibitors or placebo. IV, inverse variance**

**Figure S6. Comparison of HiSCR50 between patients receiving IL-17 inhibitors or placebo according to treatment frequency. IV, inverse variance**
